# A matrix-based approach to solving the inverse Frobenius–Perron problem using sequences of density functions of stochastically perturbed dynamical systems

**DOI:** 10.1016/j.cnsns.2017.05.011

**Published:** 2018-01

**Authors:** Xiaokai Nie, Daniel Coca

**Affiliations:** a Department of Automatic Control and Systems Engineering, The University of Sheffield, Sheffield S1 3JD, United Kingdom; b Leeds Institute for Data Analytics, University of Leeds, Leeds LS2 9JT, United Kingdom

**Keywords:** Nonlinear systems, Chaotic maps, Probability density functions, Inverse Frobenius–Perron problem

## Abstract

The paper introduces a matrix-based approach to estimate the unique one-dimensional discrete-time dynamical system that generated a given sequence of probability density functions whilst subjected to an additive stochastic perturbation with known density.

## Highlights

• The paper introduces new method of reconstructing an unknown one-dimensional transformation that is subject to constantly applied stochastic perturbations based on temporal sequences of probability density functions.
• The main assumption is that the one-dimensional transformation that generated the densities is piecewise-linear, semi-Markov.
• A matrix approximation of the transfer operator associated with the stochastically perturbed transformation, which forms the basis for the reconstruction algorithm, is introduced.
• A practical algorithm to estimate the matrix-representation of the Frobenius-Perron operator associated with the unperturbed transformation and reconstruct the onedimensional map is proposed.
• The algorithm is extended to nonlinear continuous maps.
• Numerical simulation examples are provided to demonstrate the performance of the approach and to compare it with that of an existing algorithm.

## 1 Introduction

A dynamical system, whose evolution is completely dictated by deterministic equations, can under certain conditions exhibit chaotic behavior and generate a density of states [1]. Chaotic behavior has been observed in many real-world systems including biological, physical and economic systems [2], [3], [4]. The simplest dynamical systems that exhibit chaos are one-dimensional maps. Such one-dimensional discrete dynamical systems are used to describe the evolution of many real-world systems including olfactory systems [5], electrical circuits [6], communication networks [7], rotary drills [8], chemical reactions [9] and the heart [10].

An important challenge is to develop such model from experimental observations [11], [12], [13]. Conventional approaches [14], [15], [16] rely on time series data. However, often it is not possible to measure point trajectories. For example, particle image velocimetry, a technique used to generate instantaneous velocity measurements in fluids, identifies individual tracer particles in consecutive images captured at high speeds but cannot resolve their individual orbits [17]. In such cases, in the absence of individual point trajectories, it is desirable to determine the underlying dynamical system that generated the observed density functions. Given a non-singular transformation, the evolution of an initial density function under the action of the transformation is described by the Frobenius–Perron operator associated with the transformation [18]. The fixed point of such an operator represents the invariant density under the transformation. The problem of inferring a dynamical system, whose invariant density function is given, is known as the Inverse Frobenius–Perron problem [19].

In general, solving the inverse problem involves deriving a finite-dimensional representation of the operator, which is then used to construct the dynamical system. Ulam conjectured [20] that a general infinite-dimensional Frobenius–Perron operator can be approximated by a finite rank Markov operator. For one-dimensional transformations Li [21] has shown that given a sequence of piecewise constant approximations *P_n_* of the Frobenius–Perron operator *P*, the corresponding sequence of fixed points *f_n_* of *P_n_* converge to the invariant density (i.e. fixed point) of the operator, thus proving Ulam's conjecture. In this context, the problem of determining the dynamical system that corresponds to the finite dimensional approximation of the Frobenius–Perron operator is also known as the inverse Ulam problem [22], [23].

A numerical algorithm to determine a one-dimensional transformation given the invariant density function was proposed in [19]. The algorithm however does not provide an explicit relationship between the invariant density of the one-dimensional map and the map itself. In [24] a graph-theoretic approach is introduced to construct piecewise-linear transformations that possess piecewise-constant invariant density functions that have value 0 in all relative minima points.

A generalization of these methods is presented in [25] which introduces a relationship between any arbitrary piecewise density function and a semi-Markov piecewise linear transformation defined over a partition of the interval of interest. This forms the basis for a matrix-based method to reconstruct a 3-band transformation, a special class of semi-Markov transformations, which has a given piecewise-constant density function as invariant density. The inverse problem was studied in [26] for a class of symmetric maps that have invariant symmetric Beta density functions and the unique solution can be achieved under given symmetry constraints. This method was generalized in [27] which considers a broader class of continuous unimodal maps for which each branch of the map covers the complete interval and the invariant densities are a asymmetric beta functions. Given arbitrary invariant densities similar approaches were proposed for identifying the maps with specified forms: two types of one-dimensional symmetric maps [28], smooth chaotic map with closed form [29], [30], multi-branches complete chaotic map [31]. Problems of synthesizing one-dimensional maps with prescribed invariant density function or autocorrelation function were considered in [32], [33]. Using positive matrix theory an approach to synthesizing chaotic maps with arbitrary piecewise constant invariant densities and arbitrary mixing properties was developed in [34]. This method was further extended to synthesizing dynamical systems with desired statistical properties [35], developing communication networks [36] and designing randomly switched chaotic maps and two-dimension chaotic maps used for image generation [37]. The global, open-loop strategy to control chaos proposed in [22], [23] is formulated as an inverse Frobenius–Perron problem. The aim is to perturb the original dynamical system to achieve the desired invariant measure. This reduces to the problem of finding a perturbation of the original Frobenius–Perron matrix to achieve the target invariant density function and then solving the inverse Ulam problem to determine the perturbed dynamical system.

In general, the solution to the inverse Frobenius–Perron problem is not unique. Different transformations exhibiting strikingly different dynamics may share the same invariant density functions. Additional limiting assumptions or constraints are required to ensure uniqueness of the solution [26], [27], [28], [29], [30], [31], [32], [33], [34]. In [38] a new method is proposed to solve the inverse Frobenius–Perron problem based not only on the invariant density function but also on sequences of probability density functions generated by the transformation, which ensures uniqueness of the solution. The method has been shown to be quite robust to noise. For small levels of noise this is indeed expected in light of the convergence results for noise perturbed systems established by Bollt et al. [39]. However, the accuracy of the reconstruction starts to deteriorate significantly above a certain level of noise. For large levels of noise, the approximation errors can be drastically reduced by taking into account the density function of the noise, which is often known *a priori* or can be estimated.

This paper proposes a new method to estimate the piecewise linear and expanding semi-Markov transformation that generated a temporal sequences of probability density functions whilst subjected to constantly applied stochastic perturbations. The method is extended to more general nonlinear transformations that can be approximated arbitrarily close by piecewise linear functions.

To differentiate with the normal deterministic inverse problem, we call this the inverse stochastic Frobenius–Perron problem. The emphasis here is on recovering the unknown transformation that generated the sequence of densities rather than one of the many possible transformation that share the same invariant density function.

To this end, we formulate the matrix representation of the transfer operator associated with the stochastically perturbed system in terms of the Frobenius–Perron matrix associated with the unperturbed system that we aim to estimate. This representations forms the basis for a the proposed algorithm to estimate the ‘unperturbed’ Frobenius–Perron matrix from sequences of probability density functions generated by the unknown, stochastically perturbed dynamical system, under the assumption that the density function of the perturbation is known. For general nonlinear transformations, we present a practical method to solve the inverse Ulam problem, which allows determining the sign of the derivative for each interval of the partition.

Whilst the sign of the derivative is not important when the goal is to determine a transformation that has a given invariant density function [22], this step is crucial if the aim is to reconstruct/approximate the true dynamical system that generated the data. We demonstrate that the proposed approach can reconstruct the underlying dynamical system that is subject to stochastic perturbations.

This paper is organized as follows. Section 2 introduces the inverse stochastic Frobenius–Perron problem. A matrix approximation of the transfer operator associated with the stochastically perturbed transformation is derived in Section 3. Section 4 introduces a methodology for reconstructing piecewise-linear semi-Markov transformations subject to stochastic perturbations, from sequences of density functions. The approach is extended in Section 5 to general nonlinear maps. Section 6 presents two numerical simulation examples that demonstrate the significant improvement in reconstruction accuracy achieved by the proposed algorithm that incorporates a priori knowledge of the noise in the reconstruction of the unknown transformation. Conclusions are given in Section 7.

## 2 Description of the inverse problem

Let R=[0,b], B be a Borel *σ*-algebra of subsets in *R*, and *μ* denote the normalized Lebesgue measure on *R*. Let *S: R* → *R*be a measurable, non-singular transformation, that is, $\mu (S^{-1}\left(A\right))\in B$ for any A∈B and $\mu (S^{-1}\left(A\right))=0$ for all A∈B with μ(A)=0. If *x_n_* is a random variable on *R* having the probability density function $f_{n}\in D\left(R,B,\mu \right)$, $D=\{f\in L^{1}\left(R,B,\mu \right):f\geq 0,{∥f∥}_{1}=1\}$, such that

(1) $$\text{Prob}\left\{x_{n}\in A\right\}=\int_{A}f_{n}\;d\mu .$$

It follows that $x_{n+1}$ given by

(2) $$x_{n+1}=S\left(x_{n}\right),$$

is distributed according to the probability density function $f_{n+1}=P_{S}f_{n}$, where *P_S_: L*^1^(*R*) → *L*^1^(*R*), defined by

(3) $$\int_{A}P_{S}f_{n}d\mu =\int_{S^{-1}\left(A\right)}f_{n}d\mu ,$$

is the Frobenius–Perron operator [1] associated with the unperturbed transformation *S*.

If *A* = [*a, x*],*P_S_* can be written explicitly as

(4) $$f_{n+1}\left(x\right)=P_{S}f_{n}\left(x\right)=\frac{d}{dx}\int_{S^{-1}\left(\left[a,x\right]\right)}f_{n}d\mu ,$$

Let $ℜ=\{R_{1},R_{2},\ldots ,R_{N}\}$ be a partition of *R* into intervals, and $\text{int}\left(R_{i}\right)\cap \text{int}\left(R_{j}\right)=\emptyset$ if *i* ≠ *j*. Assuming that *S* is piecewise monotonic and expanding [18],

(5) $$P_{S}f_{n}\left(x\right)=\sum_{i=1}^{N}\frac{f_{n}\left(S_{i}^{-1}\left(x\right)\right)}{\left|S^{\prime }\left(S_{i}^{-1}\left(x\right)\right)\right|}\chi_{S(R_{i})}\left(x\right),$$

where *S_i_* is the monotonic restriction of *S* on the interval *R_i_*.

A more complicated situation arises when the dynamical system is subjected to an additive random perturbation [1] such that

(6) $$x_{n+1}=\bar{S}\left(x_{n},\xi_{n}\right)=S\left(x_{n}\right)+\xi_{n},$$

where *S: R* → *R* is a given transformation and $\xi_{1},\xi_{2},\ldots ,\xi_{n}$ are independent random variables. The ‘stochastic’ Frobenius–Perron operator $\bar{P}$ corresponding to the perturbed dynamical systems is defined by [1], [39]

(7) $$\bar{P}f\left(x\right)=\int_{R}\tau (x,y)f(y)dy,$$

where τ(x,y)=g(x−S(y))is a stochastic kernel, satisfying *τ*(*x, y*) > 0, and $\int_{R}\tau (x,y)=1$.

Here, we consider the deterministic system with constantly applied stochastic perturbations

(8) $$x_{n+1}=S\left(x_{n}\right)+\xi_{n}(\text{mod}\;b),$$

where *S*: [0, *b*] → [0, *b*] is a piecewise monotonic and expanding transformation, *ξ_n_* is an independent random variable with a probability density function *g* that has compact support on [−ɛ,ɛ], i.e. *ξ_n_* is bounded in [−ɛ,ɛ], ɛ ≤ *b*.

For an arbitrary Borel set *B*⊂[0, *b*], the probability of $x_{n+1}$ falling into *B* is given by

(9) $$\text{Prob}\left\{x_{n+1}\in B\right\}=\int_{R}\int_{\left[-ɛ,ɛ\right]}f_{n}\left(x\right)g\left(\xi \right)dxd\xi ,$$

for $x_{n+1}=\bar{S}\left(x_{n},\xi_{n}\right)$, where $\bar{S}\left(x_{n},\xi_{n}\right)=S\left(x_{n}\right)+\xi_{n}\left(\;\mathrm{mod}\;\;b\right)$.

Let y=S(x)+ξ(modb). It follows that

(10) $$y=S\left(x\right)+\xi -b\chi_{\left(b,b+ɛ\right]}\left(S\left(x\right)+\xi \right)+b\chi_{\left[-ɛ,0\right)}\left(S\left(x\right)+\xi \right),$$

and

(11) $$\xi =y-S\left(x\right)+b\chi_{\left(-b,ɛ-b\right]}\left(y-S\left(x\right)\right)-b\chi_{\left[b-ɛ,b\right)}\left(y-S\left(x\right)\right),$$

then, (9) is rewritten as

(12) $$\begin{array}{ccc} \text{Prob}\{x_{n+1}\in B\} & = & \int_{B}f_{n+1}\left(x\right)dx\\ & = & \int_{B}\int_{R}f_{n}\left(x\right)g\left(y-S\left(x\right)+b\chi_{\left(-b,ɛ-b\right]}\left(y-S\left(x\right)\right)-b\chi_{\left[b-ɛ,b\right)}\left(y-S\left(x\right)\right)\right)dydx, \end{array}$$

where $f_{n+1}$ is the probability density function of $x_{n+1}$. The stochastic Frobenius–Perron operator $\bar{P}:L^{1}\left(R\right)\to L^{1}\left(R\right)$, associated with the perturbed transformation (8), is then defined by

(13) $$\begin{array}{ccc} \bar{P}f_{n}\left(x\right) & = & f_{n+1}\left(x\right)\\ & = & \int_{R}f_{n}\left(z\right)g\left(x-S\left(z\right)+b\chi_{\left(-b,ɛ-b\right]}\left(x-S\left(z\right)\right)-b\chi_{\left[b-ɛ,b\right)}\left(x-S\left(z\right)\right)\right)\;dz. \end{array}$$

It is easy to see that for any *ξ_n_* there are *N*_1_ ≤ *N* disjoint intervals ${\left\{I_{\xi_{n}}^{i}\right\}}_{i=1}^{N_{1}}$ such that for $x_{n}\in I_{\xi_{n}}^{i}$, $i=1,\ldots ,N_{1}$, *S*(*x_n_*) is monotonic and $S\left(x_{n}\right)+\xi_{n}\in \left[b,b+ɛ\right]$ or $S\left(x_{n}\right)+\xi_{n}\in \left[-ɛ,0\right]$ (i.e. maps outside the interval [0, *b*]), and *N*_2_ ≤ *N* disjoint intervals ${\left\{{\bar{I}}_{\xi_{n}}^{i}\right\}}_{i=1}^{N_{2}}$ such that for $x_{n}\in {\bar{I}}_{\xi_{n}}^{j}$, $j=1,\ldots ,N_{2}$, *S*(*x_n_*) is monotonic and $S\left(x_{n}\right)+\xi_{n}\in \left[0,b\right]$.

We have ${\bar{I}}_{\xi_{n}}^{j}\cap {\bar{I}}_{\xi_{n}}^{i}=\emptyset$, ∀*i* ≤ *N*_1_, *j* ≤ *N*_2_, and $\left(\cup_{i=1}^{N_{1}}I_{\xi_{n}}^{i}\right)\cup \left(\cup_{j=1}^{N_{2}}{\bar{I}}_{\xi_{n}}^{j}\right)=\left[0,b\right]$. For each integers *i* ≤ *N*_1_ and *j* ≤ *N*_2_, there exist unique integers *α*(*i*) ≠ *β*(*j*) ≤ *N* such that $I_{\xi_{n}}^{i}\subset R_{\alpha (i)}$ and ${\bar{I}}_{\xi_{n}}^{j}\subset R_{\beta (j)}$. From (13) it follows that

(14) $$\begin{array}{ccc} \bar{P}f_{n}\left(x\right) & = & \sum_{i=1}^{N_{1}}\int_{I_{\xi_{n}}^{i}}f_{n}\left(z\right)g\left(x-S\left(z\right)+b\chi_{\left(-b,ɛ-b\right]}\left(x-S\left(z\right)\right)-b\chi_{\left[b-ɛ,b\right)}\left(x-S\left(z\right)\right)\right)\;dz\\ & & +\;\sum_{j=1}^{N_{2}}\int_{{\bar{I}}_{\xi_{n}}^{j}}f_{n}\left(z\right)g\left(x-S\left(z\right)\right)\;dz. \end{array}$$

Substituting y=S(z) gives

(15) $$\begin{array}{ccc} \bar{P}f_{n}\left(x\right) & = & \sum_{i=1}^{N_{1}}\int_{I_{\xi_{n}}^{i}}\frac{f_{n}\left(S^{-1}\left(y\right)\right)}{S^{\prime }\left(S^{-1}\left(y\right)\right)}\chi_{S(I_{\xi_{n}}^{i})}\left(y\right)g\left(x-y+b\chi_{\left(-b,ɛ-b\right]}\left(x-y\right)-b\chi_{\left[b-ɛ,b\right)}\left(x-y\right)\right)\;dy\\ & & +\;\sum_{j=1}^{N_{2}}\int_{{\bar{I}}_{\xi_{n}}^{j}}\frac{f_{n}\left(S^{-1}\left(y\right)\right)}{S^{\prime }\left(S^{-1}\left(y\right)\right)}\chi_{S({\bar{I}}_{\xi_{n}}^{j})}\left(y\right)g\left(x-y\right)\;dy. \end{array}$$

We have

(16) $$\begin{array}{ccc} & & \sum_{i=1}^{N_{1}}\int_{I_{\xi_{n}}^{i}}\frac{f_{n}\left(S^{-1}\left(y\right)\right)}{S^{\prime }\left(S^{-1}\left(y\right)\right)}\chi_{S(I_{\xi_{n}}^{i})}\left(y\right)\;dy+\sum_{j=1}^{N_{2}}\int_{{\bar{I}}_{\xi_{n}}^{j}}\frac{f_{n}\left(S^{-1}\left(y\right)\right)}{S^{\prime }\left(S^{-1}\left(y\right)\right)}\chi_{S({\bar{I}}_{\xi_{n}}^{j})}\left(y\right)\;dy\\ & & \;=\sum_{k=1}^{N^{\prime }}\int_{{\tilde{I}}_{\xi_{n}}^{k}}\frac{f_{n}\left(S^{-1}\left(y\right)\right)}{S^{\prime }\left(S^{-1}\left(y\right)\right)}\chi_{S({\tilde{I}}_{\xi_{n}}^{k})}\left(y\right)\;dy. \end{array}$$

where ${\tilde{I}}_{\xi_{n}}^{k}\in$$\left\{I_{\xi_{n}}^{1},\ldots ,I_{\xi_{n}}^{N_{1}},{\bar{I}}_{\xi_{n}}^{1},\ldots ,{\bar{I}}_{\xi_{n}}^{N_{2}}\right\}$. Since ${\left\{S\left(I_{\xi_{n}}^{i}\right)\right\}}_{i=1,\ldots ,N_{1}}\cup {\left\{S\left({\bar{I}}_{\xi_{n}}^{j}\right)\right\}}_{i=1,\ldots ,N_{2}}=R$, the following equality holds

(17) $$\sum_{k=1}^{N^{\prime }}\int_{{\tilde{I}}_{\xi_{n}}^{k}}\left[\frac{f_{n}\left(S^{-1}\left(y\right)\right)}{S^{\prime }\left(S^{-1}\left(y\right)\right)}\chi_{S({\tilde{I}}_{\xi_{n}}^{k})}\left(y\right)\right]dy=\int_{R}\sum_{i=1}^{N}\left[\frac{f_{n}\left(S^{-1}\left(y\right)\right)}{S^{\prime }\left(S^{-1}\left(y\right)\right)}\chi_{S(R_{i})}\left(y\right)\right]dy.$$

It follows that

(18) $$\begin{array}{ccc} \bar{P}f\left(x\right) & = & \int_{R}g(x-y+b\chi_{\left(-b,\;ɛ-b\right]}\left(x-y\right)-b\chi_{\left[b-ɛ,\;b\right)}\left(x-y\right))\\ & & \cdot \sum_{i=1}^{N}\left[\frac{f_{n}\left(S^{-1}\left(y\right)\right)}{S^{\prime }\left(S^{-1}\left(y\right)\right)}\chi_{S(R_{i})}\left(y\right)\right]dy. \end{array}$$

So that

(19) $$\bar{P}f\left(x\right)=\int_{R}g(x-y+b\chi_{\left(-b,\;ɛ-b\right]}\left(x-y\right)-b\chi_{\left[b-ɛ,\;b\right)}\left(x-y\right))P_{S}f_{n}\left(y\right)dy.$$

Eq. (19) provides the link between the operator $\bar{P}$corresponding to the randomly perturbed dynamical system (8) and the Frobenius–Perron operator *P_S_* associated to the noise-free system. The inverse problem is formulated as follows.

Let ${\left\{x_{0,i}^{j}\right\}}_{i,j=1}^{\theta ,K}$ and ${\left\{x_{1,i}^{j}\right\}}_{i,j=1}^{\theta ,K}$ be *K* sets of initial and final state observations, respectively, such that

(20) $$x_{1,i}^{j}=S\left(x_{0,i}^{j}\right)+\xi_{0}^{j}\;(\text{mod}\;\;\;b),\;\;j=1,\ldots ,K$$

It is assumed that the measurement system does not allow associating an initial state $x_{0,i}^{j}$ with its image $x_{1,i}^{j}$ under the transformation. The inverse problem considered here is to determine the transformation *S* in (8) given the noise density function *g* and probability density functions ${\left\{f_{0}^{j}\right\}}_{j=1}^{K}$, ${\left\{f_{1}^{j}\right\}}_{j=1}^{K}$ associated with the initial states${\left\{x_{0,i}^{j}\right\}}_{i,j=1}^{\theta ,K}$ and final states ${\left\{x_{1,i}^{j}\right\}}_{i,j=1}^{\theta ,K}$ , that is$f_{1}^{j}=\bar{P}f_{0}^{j}$, j=1,…,K, where$\bar{P}$ is the transfer operator associated with perturbed transformation (8).

## 3 A matrix representation of the transfer operator $\bar{P}$

Let *S* be a piecewise linear and expanding semi-Markov transformation over the *N*-interval partition, $ℜ=\{R_{1},\;R_{2},\;\ldots ,\;R_{N}\}$.

Definition 1 A transformation *S: R* → *R* is said to be semi-Markov with respect to the partition ℜ (or ℜ-semi-Markov) if there exist disjoint intervals $Q_{j}^{\left(i\right)}$ so that $R_{i}=\cup_{k=1}^{p(i)}Q_{k}^{\left(i\right)}$, i=1,…,N, the restriction of *S* to $Q_{k}^{\left(i\right)}$, denoted ${S|}_{Q_{k}^{\left(i\right)}}$, is monotonic and $S(Q_{k}^{\left(i\right)})\in ℜ$. [25]

The restriction ${S|}_{R_{i}}$is a homeomorphism from *R_i_* to a union of intervals of ℜ

(21) $$\overset{p(i)}{\underset{k=1}{⋃}}R_{r(i,k)}=\overset{p(i)}{\underset{k=1}{⋃}}S(Q_{k}^{\left(i\right)}),$$

where $R_{r(i,k)}=S\left(Q_{k}^{\left(i\right)}\right)\in ℜ$, $Q_{k}^{\left(i\right)}=\left[q_{k-1}^{\left(i\right)},q_{k}^{\left(i\right)}\right]$, i=1,…,N,k=1,…,p(i) and *p*(*i*) denotes the number of disjoint subintervals $Q_{k}^{\left(i\right)}$ corresponding to *R_i_*.

Let *f_n_* be a piecewise constant function over the partition *R* such that $f_{n}\left(x\right)=$$\sum_{i=1}^{N}w_{i}\chi_{R_{i}}\left(x\right)$. Its image under transformation *P_S_f_n_* is also a piecewise constant function over ℜ [18] such that $P_{S}f_{n}\left(x\right)=\sum_{i=1}^{N}u_{i}\chi_{R_{i}}\left(x\right)$ and the Frobenius–Perron operator can be represented by a finite-dimensional matrix

(22) $$P_{S}f_{n}\left(x\right)=\sum_{j=1}^{N}\left(\sum_{i=1}^{N}\left(w_{i}m_{i,j}\right)\right)\chi_{R_{j}}\left(x\right),$$

where $M={\left(m_{i,j}\right)}_{1\leq i,j\leq N}$ is the Frobenius–Perron matrix induced by *S* with entries given by

(23) $$m_{i,j}=\left\{ \begin{array}{cc} {\left|{\left({S|}_{Q_{j}^{\left(i\right)}}\right)}^{\prime }\right|}^{-1}, & \text{if}\;S\left(Q_{k}^{\left(i\right)}\right)=R_{j};\\ 0, & \text{otherwise}. \end{array}\right.$$

From (22) it follows that

(24) $$u_{j}=\sum_{i=1}^{N}w_{i}m{}_{i,j},$$

for j=1,…,N.

Let $\bar{ℜ}=\left\{{\bar{R}}_{1},\;{\bar{R}}_{2},\;\ldots ,\;{\bar{R}}_{N}\right\}$ be a regular partition of *R* into *N* equal sized intervals. By integrating (19) over an interval ${\bar{R}}_{k}\in \bar{ℜ}$ gives

(25) $$\int_{{\bar{R}}_{k}}\bar{P}f_{n}\left(x\right)\;dx=\int_{{\bar{R}}_{k}}\int_{I}g\left(x-y+b\chi_{\left(-b,\;ɛ-b\right]}\left(x-y\right)-b\chi_{\left[b-ɛ,\;b\right)}\left(x-y\right)\right)\cdot P_{S}f_{n}\left(y\right)\;dydx$$

Consider the following approximation

(26) $$\begin{array}{ccc} \bar{P}f_{n}\left(x\right) & = & p_{N}\left(x\right)+q_{N}\left(x\right)\\ & = & \sum_{k=1}^{N}v_{k}\chi_{{\bar{R}}_{k}}\left(x\right)+q_{N}\left(x\right), \end{array}$$

where $p_{N}\left(x\right)={\bar{P}}_{N}f_{n}\left(x\right)$ is the orthogonal projection of $\bar{P}f_{n}$ in *L*^1^ on the finite-dimensional space spanned by, *q_N_*(*x*) is the orthogonal complement in *L*^1^ and

(27) $$v_{k}=\frac{1}{\lambda ({\bar{R}}_{k})}\int_{{\bar{R}}_{k}}\int_{I}g\left(x-y+b\chi_{\left(-b,\;ɛ-b\right]}\left(x-y\right)-b\chi_{\left[b-ɛ,\;b\right)}\left(x-y\right)\right)\cdot P_{S}f_{n}\left(y\right)\;dydx,$$

where $\lambda ({\bar{R}}_{k})$ is the Lebesgue measure on ${\bar{R}}_{k}$. Clearly, *q_N_*(*x*) → 0 as N→+∞.

It follows that

(28) $$\begin{array}{ccc} v_{k} & = & \frac{1}{\lambda ({\bar{R}}_{k})}\int_{{\bar{R}}_{k}}\sum_{j=1}^{N}\left[\int_{R_{j}}g\left(x-y+b\chi_{\left(-b,\;ɛ-b\right]}\left(x-y\right)-b\chi_{\left[b-ɛ,\;b\right)}\left(x-y\right)\right)\;dy\cdot u_{j}\right]\;dx\\ & = & \frac{N}{b}\sum_{j=1}^{N}\left[\int_{{\bar{R}}_{k}}\int_{R_{j}}g\left(x-y+b\chi_{\left(-b,\;ɛ-b\right]}\left(x-y\right)-b\chi_{\left[b-ɛ,\;b\right)}\left(x-y\right)\right)\;dydx\cdot u_{j}\right]. \end{array}$$

Let $D={\left(d_{k,j}\right)}_{1\;\leq \;k\;\leq \;N;\;1\;\leq \;j\;\leq \;N}$ be the *N* × *N* matrix with entries given by

(29) $$d_{k,j}=\frac{N}{b}\int_{{\bar{R}}_{k}}\int_{R_{j}}g(x-y+b\chi_{\left(-b,\;ɛ-b\right]}\left(x-y\right)-b\chi_{\left[b-ɛ,\;b\right)}\left(x-y\right))\;dydx.$$

Substituting (24), (29) in (28) leads to

(30) $$v^{f_{n+1}}=w^{f_{n}}\cdot M\cdot D^{\prime }=w^{f_{n}}\cdot Q,$$

where $w^{f_{n}}=\left[w_{1},\ldots ,w_{N}\right]$, $v^{f_{n+1}}=\left[v_{1},\ldots ,v_{N}\right]$ are the coefficient vectors associated with the piecewise constant density functions *f_n_* and $\bar{P}f_{n}$ respectively and $Q=M\cdot D^{\prime }$ is the matrix approximation of the operator $\bar{P}$. Eq. (30) maps a piecewise-constant density function over the *N*-dimensional partition, which in general is non-uniform, to a piecewise-constant density function over a uniform *N*-dimensional partition. Eq. (30) is the basis for the new algorithm to reconstruct the transformation *S* given pairs of successive density functions generated by the stochastically perturbed transformation.

In practice, we can chose a finer *N*_1_-interval partition $\bar{ℜ}$, *N*_1_ >> *N*. For example, we can construct $\bar{ℜ}$ as a refinement of the partition ℜ such that the cut points of partition ℜ are a subset of cut-points associated with $\bar{ℜ}$. This leads to an alternative formulation of (30) where both the initial and final densities are defined over the same partition. Given an initial piecewise density function *f* over the partition$\bar{ℜ}$, the matrix approximation can then be used to compute a sequence of successive iterations by the corresponding finite-dimensional approximation of the stochastic Frobenius–Perron operator.

## 4 Solving the inverse stochastic Frobenius–Perron problem for piecewise linear semi-Markov transformations

This section presents an approach to solving the inverse stochastic Frobenius–Perron problem, under the assumption that *S*: [0, *b*] → [0, *b*] is a piecewise linear semi-Markov transformation over a partition ℜ,

(31) $$\begin{array}{ccc} ℜ & = & \left\{R_{1},R_{2},\ldots ,R_{N}\right\}\\ & = & \{\left[0,a_{1}\right],\left(a_{1},a_{2}\right],\ldots ,\left(a_{N-1},a_{N}\right]\}, \end{array}$$

$a_{N}=b$, which is assumed to be known. In what follows we assume that $\bar{ℜ}$ is defined as the uniform partition of dimension *N* of [0, *b*].

The main steps of the approach are summarized below:

*Step* 1: Given the observations $X_{t}={\left\{x_{t,j}\right\}}_{j=1}^{\theta }$, *t* = 0,…,*T*, estimate the coordinate vectors $w^{f_{t}}=\left[w_{t,1},\ldots ,w_{t,N}\right]$ and $v^{f_{t+1}}=\left[v_{t+1,1},\ldots ,v_{t+1,N}\right]$, *t* = 0,…,*T*−1 corresponding to the piecewise constant density functions $f_{t}\left(x\right)$ over ℜ and $f_{t+1}\left(x\right)={\bar{P}}_{S}f_{t}\left(x\right)$ over $\bar{ℜ}$, respectively. Compute the matrix ***D*** defined in (29).
*Step* 2: Estimate ***M***, the matrix representation of the Frobenius–Perron operator *P_S_* associated with the deterministic transformation *S*.
*Step* 3: Construct the piecewise linear semi-Markov transformation over ℜ.
  These steps are described below in more detail.

### 4.1 Step 1: estimate ***w*** and ***v*** and compute ***d***

Let *f*_0_(*x*) be an initial density function that is piecewise constant on the partition$ℜ=\{R_{1},\ldots ,R_{N}\}$.

(32) $$f_{0}\left(x\right)=\sum_{i=1}^{N}w_{0,i}\chi_{R_{i}}\left(x\right),$$

where the coefficients satisfy $\sum_{i=1}^{N}w_{0,i}\lambda \left(R_{i}\right)=1$. Let $X_{0}={\left\{x_{0,j}\right\}}_{j=1}^{\theta }$ be the set of initial states obtained by sampling *f*_0_(*x*). The states $X_{t}={\left\{x_{t,j}\right\}}_{j=1}^{\theta }$ at a given sampling time *t* > 0 are assumed to be generated by applying *t* times the process defined in (8), where $\Omega ={\left\{\xi_{i}\right\}}_{i=1}^{\theta }$ are generated by sampling *g*(*ξ*).

The density function *f_t_*(*x*) on $\bar{ℜ}$ associated with the states *X_t_* is given by

(33) $$f_{t}\left(x\right)=\sum_{i=1}^{N}v_{t,i}\chi_{{\bar{R}}_{i}}\left(x\right),$$

where the coefficients $v_{t,i}=\frac{1}{\lambda ({\bar{R}}_{i})\cdot \theta }\sum_{j=1}^{\theta }\chi_{{\bar{R}}_{i}}\left(x_{t,j}\right)=\frac{N}{b\theta }\sum_{j=1}^{\theta }\chi_{{\bar{R}}_{i}}\left(x_{t,j}\right)$. In practice the densities *f_t_*(*x*) are estimated directly from observations.

We define the following matrices

(34) $$W_{0}=\left[\begin{array}{c} w^{f_{0}}\\ w^{f_{1}}\\ \vdots \\ w^{f_{T-1}} \end{array}\right]=\left[\begin{array}{cccc} w_{0,1} & w_{0,2} & \cdots & w_{0,N}\\ w_{1,1} & w_{1,2} & \cdots & w_{2,N}\\ \vdots & \vdots & \ddots & \vdots \\ w_{T-1,1} & w_{T-1,2} & \cdots & w_{T-1,N} \end{array}\right],$$

and

(35) $$V=\left[\begin{array}{c} v^{f_{1}}\\ v^{f_{2}}\\ \vdots \\ v^{f_{T}} \end{array}\right]=\left[\begin{array}{cccc} v_{1,1} & v_{1,2} & \cdots & v_{1,H}\\ v_{2,1} & v_{2,2} & \cdots & v_{2,H}\\ \vdots & \vdots & \ddots & \vdots \\ v_{T,1} & v_{T,2} & \cdots & v_{T,H} \end{array}\right].$$

The matrix ***D*** is obtained by numerical integration of (29).

### 4.2 Step 2: estimate the Frobenius–Perron matrix ***m***

This is carried out in two stages. Firstly, the coordinate vector corresponding to the piecewise constant densities $P_{S}f_{t}\left(x\right)=\sum_{i=1}^{N}u_{t,i}\chi_{R_{i}}\left(x\right)$ over the partition ℜ are obtained by solving the following constrained optimization problem

(36) $$\min_{0\leq {\left\{u_{t,j}\right\}}_{t=0,\ldots ,T-1,j=1,\ldots ,N}\leq N}{∥V-Y\cdot D^{\prime }∥}_{F},$$

subject to

(37) $$\sum_{i=1}^{N}u_{t,i}\lambda \left(R_{i}\right)=1,\;\text{for}\;\;t=0,\ldots ,T-1.$$

where

(38) $$Y=\left[\begin{array}{c} u^{f_{0}}\\ u^{f_{1}}\\ \vdots \\ u^{f_{T-1}} \end{array}\right]=\left[\begin{array}{cccc} u_{0,1} & u_{0,2} & \cdots & u_{0,N}\\ u_{1,1} & u_{1,2} & \cdots & u_{2,N}\\ \vdots & \vdots & \ddots & \vdots \\ u_{T-1,1} & u_{T-1,2} & \cdots & u_{T-1,N} \end{array}\right],$$

and || · ||_*F*_ denotes the Frobenius norm.

In the second stage, the matrix representation of the Frobenius–Perron operator associated with the unperturbed transformation *S* is obtained as a solution to the following constrained optimization problem

(39) $$\min_{{\left\{m_{i,j}\right\}}_{i,j=1}^{N}\geq 0}∥Y-W_{0}M|{|}_{F},$$

subject to

(40) $$\sum_{j=1}^{N}m_{i,j}\lambda \left(R_{j}\right)=\lambda \left(R_{i}\right),\;\;\;\;\text{for}\;i=1,\ldots ,N.$$

In the following it is shown that the matrices $\Phi_{1}=D^{\prime }D$ and $\Phi_{2}={W^{\prime }}_{0}W_{0}$ are non-singular, which ensures uniqueness of solutions.

Proposition 1 *For a piecewise linear* ℜ*-semi-Markov transformation S subjected to additive perturbation, where*$ℜ=\bar{ℜ}$ *is an N-dimensional regular partition of* [*0, b*]*, the matrix*$\Phi_{1}=D^{\prime }D$ *is non-singular*.

Proof For $\bar{ℜ}=\left\{{\bar{R}}_{k}=R_{j}\in ℜ,k=1,\ldots ,N\right\}$, $\lambda \left(R_{j}\right)=\lambda \left({\bar{R}}_{k}\right)$, *R_j_* ∈ ℜ, $R_{k}\in \bar{ℜ}$, for j,k=1,…,N,

(41) $$d_{k,j}=\frac{N}{b}\int_{R_{k}}\int_{R_{j}}g(x-y+b\chi_{\left(-b,\;ɛ-b\right]}\left(x-y\right)-b\chi_{\left[b-ɛ,\;b\right)}\left(x-y\right))\;dydx.$$

The matrix ***D*** satisfies that

(42) $$d_{k,j}=d_{j,k},\;\;d_{j,j}=d_{k,k},\;\;\forall j,k\in \left\{1,\ldots ,N\right\}$$

and $d_{k,j}=d_{k+1,j+1}$, for 1≤k,j≤N−1.

Let $d_{i}=d_{1,i}$, for i=1,…,N. The matrix ***D*** is decomposed into two triangle matrices as follows

(43) $$D=D_{l}+D_{u},$$

where

(44) $$D_{l}=\left[\begin{array}{ccccc} d_{1}/2 & & & & \\ d_{2} & d_{1}/2 & & 0 & \\ d_{3} & d_{2} & d_{1}/2 & & \\ \cdots & \cdots & \cdots & \ddots & \\ d_{N} & d_{N-1} & d_{N-2} & \cdots & d_{1}/2 \end{array}\right],$$

(45) $$D_{u}=\left[\begin{array}{ccccc} d_{1}/2 & d_{2} & d_{3} & \cdots & d_{N}\\ & d_{1}/2 & d_{2} & \cdots & d_{N-1}\\ & & d_{1}/2 & \cdots & d_{N-2}\\ & 0 & & \ddots & \cdots \\ & & & & d_{1}/2 \end{array}\right].$$

Then, $\det\left(D_{l}\right)=\det\left(D_{u}\right)=\frac{{d_{1}}^{N}}{2^{N}}$. According to the Minkowski determinant theorem, ***D***_*l*_ and ***D***_*u*_ are both non-negative, then$\det\left(D\right)\geq \det\left(D_{l}\right)+\det\left(D_{u}\right)>0$. Hence, ***D*** and *Φ*_1_ are non-singular, and this completes the proof.

Theorem 1 *Let S*: [0, *b*] → [0, *b*] *be a piecewise linear R-semi-Markov transformation subjected to additive noise,*$ℜ=\bar{ℜ}$*. Then the matrix* ***Q*** *representing the transfer operator associated with the noisy dynamical system has* 1 *as the eigenvalue of maximum modulus and also has the unique eigenvalue of modulus* 1.

Proof For $ℜ=\bar{ℜ}$, the matrix $Q=M\cdot D^{\prime }$ is square. Let $Q={\left(q_{i,j}\right)}_{1\leq i,j\leq N}$ where

(46) $$q_{i,j}=\sum_{k=1}^{N}\left(m_{i,k}d_{j,k}\right).$$

The sum of *i*th row of ***Q*** is given by

(47) $$\sum_{j=1}^{N}q_{i,j}=\sum_{j=1}^{N}\left({\left[\begin{array}{c} m_{i,1}\\ \vdots \\ m_{i,k}\\ \vdots \\ m_{i,N} \end{array}\right]}^{\prime }\left[\begin{array}{c} d_{j1}\\ \vdots \\ d_{j,k}\\ \vdots \\ d_{j,N} \end{array}\right]\right)={\left[\begin{array}{c} m_{i,1}\\ \vdots \\ m_{i,k}\\ \vdots \\ m_{i,N} \end{array}\right]}^{\prime }\left[\begin{array}{c} d_{11}+\ldots +d_{j,1}+\ldots +d_{N,1}\\ \vdots \\ d_{1,k}+\ldots +d_{j,k}+\ldots +d_{N,k}\\ \vdots \\ d_{1,N}+\ldots +d_{j,N}+\ldots +d_{N,N} \end{array}\right].$$

The column sum of *k*th column of ***D*** is given by

(48) $$\begin{array}{ccc} \sum_{i=1}^{N}d_{i,k} & = & \sum_{i=1}^{N}\frac{N}{b}\int_{R_{i}}\int_{R_{j}}g(x-y+b\chi_{\left(-b,\;ɛ-b\right]}\left(x-y\right)-b\chi_{\left[b-ɛ,\;b\right)}\left(x-y\right))\;dydx\\ & = & \frac{N}{b}\int_{I}\int_{R_{j}}g(x-y+b\chi_{\left(-b,\;ɛ-b\right]}\left(x-y\right)-b\chi_{\left[b-ɛ,\;b\right)}\left(x-y\right))\;dydx=\frac{N}{b}\cdot \frac{b}{N}\\ & = & 1. \end{array}$$

It follows that

(49) $$\sum_{j=1}^{N}q_{i,j}=\sum_{j=1}^{N}m_{i,j}.$$

For a regular partition ℜ, $\sum_{j=1}^{N}m_{i,j}=\frac{\sum_{k=1}^{p(i)}Q_{k}^{\left(i\right)}}{\lambda (R_{i})}=1$, then $\sum_{j=1}^{N}q_{i,j}=1$. Thus, ***Q*** is row stochastic. Because ***Q*** is also a positive matrix, matrix ***Q*** has 1 as the eigenvalue of maximum modulus, and the algebraic and geometric multiplicities of this eigenvalue are 1. This concludes the proof.

Remark *.* From Theorem 1 it follows that $f^{*}={\bar{P}}_{N}f^{*}$ has a unique, no-trivial solution, where ${\bar{P}}_{N}$ is the *N*-dimensional approximation of the stochastic Frobenius–Perron operator associated with the noise perturbed piecewise linear ℜ-semi-Markov transformation (8).

Proposition 2 A *noise perturbed piecewise linear* ℜ*-semi-Markov transformation S can be uniquely reconstructed given N linearly independent piecewise constant density functions*$f_{0}^{i}$ *defined over a regular partition* ℜ*, which correspond to initial states, and the piecewise constant densities*$f_{1}^{i}={\bar{P}}_{N}f_{0}^{i}$*.*

Proof Let

(50) $$f_{0}^{i}\left(x\right)=\sum_{j=1}^{N}w_{i,j}^{0}\chi_{R_{j}}\left(x\right),\;\;i=1,\ldots ,N$$

be a set of initial piecewise constant densities over a partition ℜ and

(51) $$f_{1}^{i}\left(x\right)=\sum_{j=1}^{N}w_{i,j}^{1}\chi_{R_{j}}\left(x\right),\;\;i=1,\ldots ,N$$

be the piecewise constant densities, corresponding to the final states

From (30), we have

(52) $$W_{1}=W_{0}\cdot M\cdot D^{\prime },$$

where $W_{0}=\left[\begin{array}{c} w^{f_{0}^{1}}\\ w^{f_{0}^{2}}\\ \vdots \\ w^{f_{0}^{N}} \end{array}\right]=\left[\begin{array}{cccc} w_{1,1}^{0} & w_{1,2}^{0} & \cdots & w_{1,N}^{0}\\ w_{2,1}^{0} & w_{2,2}^{0} & \cdots & w_{2,N}^{0}\\ \vdots & \vdots & \ddots & \vdots \\ w_{N,1}^{0} & w_{N,2}^{0} & \cdots & w_{N,N}^{0} \end{array}\right]$, $W_{1}=\left[\begin{array}{c} w^{f_{1}^{1}}\\ w^{f_{1}^{2}}\\ \vdots \\ w^{f_{1}^{N}} \end{array}\right]=\left[\begin{array}{cccc} w_{1,1}^{1} & w_{1,2}^{1} & \cdots & w_{1,N}^{1}\\ w_{2,1}^{1} & w_{2,2}^{0} & \cdots & w_{2,N}^{0}\\ \vdots & \vdots & \ddots & \vdots \\ w_{N,1}^{1} & w_{N,2}^{1} & \cdots & w_{N,N}^{1} \end{array}\right]$.

Because the initial densities $f_{0}^{i}$, i=1,…,N are linearly independent, the matrix ***W***_0_ is non-singular. Moreover, from Proposition 1, ***D*** is non-singular and the Frobenius–Perron matrix is given by

(53) $$M=W_{0}^{-1}W_{1}D^{\prime -1}.$$

### 4.3 Step 3: construct the piecewise linear semi-Markov transformation over ℜ

It is assumed that each branch of the map, ${S|}_{Q_{k}^{\left(i\right)}}$, is monotonically increasing. The derivative of ${S|}_{Q_{k}^{\left(i\right)}}$ is 1/*m*_*i, j*_, the length of $Q_{k}^{\left(i\right)}$ is given by

(54) $$\lambda \left(Q_{k}^{\left(i\right)}\right)=q_{k}^{\left(i\right)}-q_{k-1}^{\left(i\right)}=m_{i,j}\lambda \left(R_{j}\right)$$

which allows computing iteratively $q_{k}^{\left(i\right)}$ for each interval *R_i_* starting with $q_{0}^{\left(i\right)}=a_{i-1}$.Then the map is given by

(55) $${S|}_{Q_{k}^{\left(i\right)}}\left(x\right)=\frac{1}{m_{i,j}}\left(x-q_{k-1}^{\left(i\right)}\right)+a_{j-1},$$

for k=1,…,p(i), *j* is the index of image *R_j_* of $Q_{k}^{\left(i\right)}$, i.e. $S\left(Q_{k}^{\left(i\right)}\right)=R_{j}$, i=1,…,N, j=1,…,N, where *m*_*i, j*_ ≠ 0.

## 5 Solving the inverse stochastic Frobenius–Perron problem for general nonlinear transformations

This section considers more general nonlinear maps that are not piecewise linear semi-Markov. Starting with Lasota and Yorke [40] who established the existence of invariant measure for piecewise monotonic transformations and Li [21] who proposed a numerical procedure to calculate the invariant density function corresponding to the invariant measure, the problem of approximating the invariant density of a transformation, which is closely linked with the problem of approximating the Frobenius–Perron operator and the transformation itself, has been studied by a number of authors [41], [42], [43], [44], [45]. In [43] Gora and Boyarsky approximated a nonsingular transformation *S,* that may have infinitely many pieces of monotonicity, by a sequence of piecewise linear functions *S_n_* and shown that the invariant density of the map *S* can be approximated arbitrarily well by densities that are invariant under finite approximations *S_n_* of *S*. In general, for continuous nonsingular transformation we have the following result [22], [46]

Theorem 2 *Let S: R* → *R be a continuous transformation and let* {*S_n_*}_*n* ≥ 1_*be a sequence of transformations converging to S in the C*^0^ *topology. Let μ_n_ be a probabilistic measure invariant under S_n_, n* *=* *1,2,…. If μ is a weak*-* *limit point of the sequence* {*μ_n_*}_*n* ≥ 1_*then μ is S*-*invariant*.

This shows that the invariant densities $f_{n}^{*}$ of successive piecewise linear approximations *S_n_* of *S* converge in a weak sense to the invariant density $f^{*}$of the original transformation as *n*→∞. This means that in practice we can estimate transition matrices to approximate arbitrarily well the Frobenius–Perron operator associated with *S*. A generalization of this result for dynamical systems subjected to additive noise is presented in [39].

Here, the goal is to estimate from sequences of density functions a piecewise linear semi-Markov approximation $\hat{S}$, defined over a uniform Markov partition $ℜ=\{R_{1},R_{2},\ldots ,R_{N}\}$of [0, *b*], $\lambda (R_{i})=b/N$, of the unknown nonlinear map *S*: [0, *b*] → [0, *b*] subjected to stochastic perturbation. It is assumed that the nonlinear map *S* has an invariant density and that it can be approximated arbitrarily well by piecewise linear functions. Unlike the control problem studied in [33], here the challenge is to estimate the unknown nonlinear transformation that generated a sequence of density functions rather than one of the many possible perturbations of the original map, which yield a desired invariant density.

The proposed identification scheme for general nonlinear maps is summarized as follows:

*Step* 1: Given the observations$X_{n}={\left\{x_{n,j}\right\}}_{j=1}^{\theta }$, n=0,…,T, estimate the coordinate vectors $w^{f_{t}}$ and $v^{f_{t+1}}$ of the piecewise constant density *f_t_*(*x*) and $f_{t+1}\left(x\right)$ defined over a regular partition $ℜ=\bar{ℜ}$ of size *N*. Compute ***D*** using the given probability density function of perturbation.

*Step* 2: Estimate the matrix ***Y*** corresponding to *P_S_f_t_*(*x*)by solving the optimization problem (36). *P_S_f_t_*(*x*) can be used to identify the Frobenius–Perron matrix associated with the unknown map;

*Step* 3: Identify a trial Frobenius–Perron matrix $\hat{M}={\left({\hat{m}}_{i,j}\right)}_{1\leq i,j\leq N}$ , $\sum_{j=1}^{N}{\hat{m}}_{i,j}=1$ by solving the constrained optimization problem (39). Since the map is continuous, this is then used to further determine the indices of consecutive positive entries of each row and in this way $\hat{M}$ is refined. Let $B^{i}=\left\{r_{s}^{i},r_{s}^{i}+1,\ldots ,r_{e}^{i}\right\}$ be the set of column indices corresponding to consecutive positive entries of *i*th row and $r_{m}^{i}\in B^{i}$satisfying

(56) $${\hat{m}}_{i,r_{m}^{i}}=\max{\left\{{\hat{m}}_{i,j}\right\}}_{j=1}^{N}.$$

Therefore, the piecewise linear ℜ-semi-Markov map associated with the refined Frobenius–Perron matrix ***M*** should satisfy that $R_{r(i,k)}=S\left(Q_{k}^{\left(i\right)}\right)\in ℜ$, where $\overset{p(i)}{\underset{k=1}{\cup }}R_{r(i,k)}$ is the image of the interval *R_i_*,i=1,…,N, $p\left(i\right)=r_{e}^{i}-r_{s}^{i}+1$ and $r\left(i,k\right)\in B^{i}$ is the column index of a positive entry on the *i*th row of ***M*** satisfying

(57) r(i,k+1)=r(i,k)+1.

for i=1,...,N,k=1,...,p(i)−1.

*Step* 4: Solve the following optimization problem to determine the Frobenius–Perron matrix ***M***

(58) $$\min_{\{m_{i,j}\}\geq 0}∥Y-W_{0}M|{|}_{F},$$

subject to

(59) $$\sum_{k=1}^{p(i)}m_{i,r(i,1)+k-1}=1.$$

and

(60) $$\left\{ \begin{array}{cc} m_{i,r(i,k)}>0, & i=1,\ldots ,N;\\ m_{i,j}=0, & j\neq r(i,k), \end{array}\right.$$

for k=1,...,p(i).

*Step* 5. Determine the monotonicity of each branch ${S|}_{Q_{k}^{\left(i\right)}}$. Let $R_{i}^{\prime }=\left[a_{r(i,1)-1},a_{r(i,p(i))}\right]$ be the image of the interval *R_i_* under the semi-Markov transformation $\hat{S}$ associated with the identified Frobenius–Perron matrix ***M***. Denote $a_{r(i,1)-1}$ as the starting point of *R*_*r*(*i*, 1)_ mapped from the subinterval $Q_{1}^{\left(i\right)}$, and *a*_*r*(*i, p*(*i*))_ as the end point of *R*_*r*(*i, p*(*i*))_, the image of the subinterval $Q_{p(i)}^{\left(i\right)}$. Let ${\bar{c}}_{i}$ be the midpoint of the image $R_{i}^{\prime }$. The sign *γ*(*i*)of ${\left\{{\hat{S}}^{\prime }\left(x\right){|}_{Q_{k}^{\left(i\right)}}\right\}}_{k=1}^{p(i)}$ is given by

(61) $$\gamma \left(i\right)=\left\{ \begin{array}{cc} -1, & \text{if}\;{\bar{c}}_{i}-{\bar{c}}_{i-1}<0;\\ 1, & \text{if}\;{\bar{c}}_{i}-{\bar{c}}_{i-1}\geq 0;\\ \gamma (i-1), & \text{if}\;{\bar{c}}_{i}={\bar{c}}_{i-1}, \end{array}\right.$$

for i=2,…,N and γ(1)=γ(2).

**Fig. 1:**
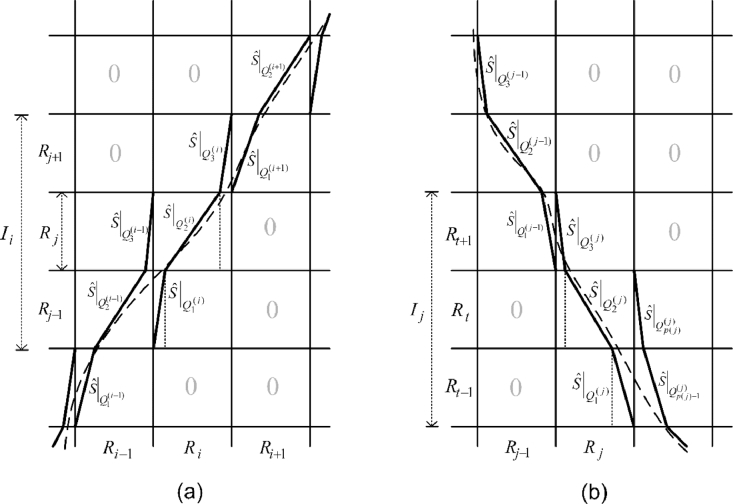
Schematic diagram of construction of piecewise linear semi-Markov transformations for monotonically increasing (a) and decreasing (b) cases. The indices of positive entries of the Frobenius–Perron matrix are determined from a trial matrix. The grayed ‘0’ in the map grid represents the corresponding entry of the refined Frobenius–Perron matrix is zero, and the other entries are positive. The dashed lines are the constructed nonlinear map $\bar{S}$ smoothed from the identified piecewise linear semi-Markov map $\hat{S}$.

*Step* 5. Construct semi-Markov map based on the Frobenius–Perron matrix ***M*** and the monotonicity of each branch. Given that the derivative of ${S|}_{Q_{k}^{\left(i\right)}}$ is 1/*m*_*i, j*_, the end point $q_{k}^{\left(i\right)}$of subinterval $Q_{k}^{\left(i\right)}$ within *R_i_* is given by

(62) $$q_{k}^{\left(i\right)}=\left\{ \begin{array}{cc} a_{i-1}+\sum_{j=1}^{k}m_{i,r(i,j)}\lambda \left(R_{r(i,j)}\right), & \text{if}\;\gamma (i)=+1;\\ a_{i-1}+\sum_{j=1}^{k}m_{i,r(i,p(i)-k+1)}\lambda \left(R_{r(i,p(i)-k+1)}\right), & \text{if}\;\gamma (i)=-1. \end{array}\right.$$

where k=1,...,p(i)−1 and $q_{p(i)}^{\left(i\right)}=a_{i}$. The piecewise linear semi-Markov transformation $\hat{S}$ on each subinterval $Q_{j}^{\left(i\right)}$ is given by

(63) $${\hat{S}|}_{Q_{j}^{\left(i\right)}}\left(x\right)=\left\{ \begin{array}{cc} \frac{1}{m_{i,j}}\left(x-q_{k-1}^{\left(i\right)}\right)+a_{j-1}, & \text{if}\;\gamma (i)=+1;\\ -\frac{1}{m_{i,j}}\left(x-q_{k-1}^{\left(i\right)}\right)+a_{j}, & \text{if}\;\gamma (i)=-1. \end{array}\right.$$

for *m*_*i, j*_ ≠ 0, i=1,…,N,j=1,…,N,k=1,...,p(i)−1. A smooth nonlinear map is obtained by fitting a polynomial smoothing spline. Fig. 1 shows the construction of monotonically increasing and decreasing piecewise linear semi-Markov transformation and the resulting smooth continuous map.

## 6 Numerical simulation studies

The proposed algorithms are demonstrated using simulated data generated by two chaotic maps.

### 6.1 Example A

Consider the noise perturbed dynamical system

(64) $$x_{n+1}=S\left(x_{n}\right)+\xi_{n},\left(\;\mathrm{mod}\;1\right)$$

where {*ξ_n_*} is white noise that follows a Gaussian distribution truncated to the range [−ɛ,ɛ] where ɛ=0.02. The piecewise linear and expanding transformation *S*: [0, 1] → [0, 1] is defined by

(65) $${S|}_{R_{i}}\left(x\right)=\alpha_{i,j}x+\beta_{i,j},$$

for i=1,…,4, j=1,…,4, over the partition $ℜ={\left\{R_{i}\right\}}_{i=1}^{4}=\{\left[0,0.3\right],\;$(0.3, 0.4], (0.4, 0.8], (0.8, 1]} where

$${\left(\alpha_{i,j}\right)}_{1\leq i,j\leq 4}=\left[\begin{array}{cccc} 3.33 & 0.83 & 13.33 & 3.33\\ 15.00 & 3.33 & 10.00 & 20.00\\ 3.75 & 2.50 & 3.33 & 1.25\\ 7.50 & 1.25 & 6.67 & 10.00 \end{array}\right],\;\;{\left(\beta_{i,j}\right)}_{1\leq i,j\leq 4}=\left[\begin{array}{cccc} 0 & 0.23 & -2.40 & 0\\ -4.50 & -0.77 & -3.10 & -7\\ -1.50 & -0.90 & -1.33 & 0\\ -6.00 & -0.75 & -5.73 & -9.00 \end{array}\right].$$

**Fig. 2:**
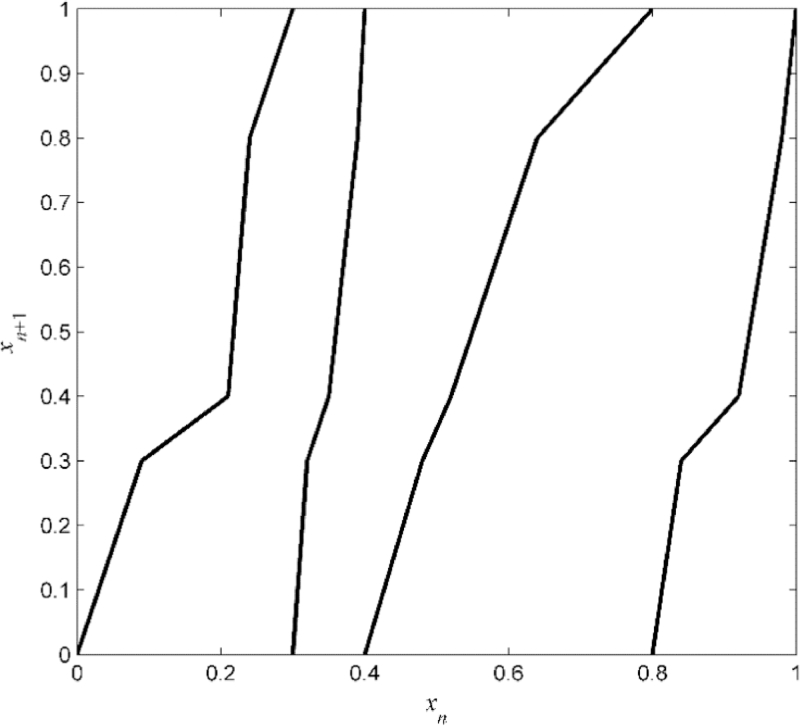
Example A: Original piecewise linear transformation *S*.

The graph of *S* is shown in Fig. 2.

**Fig. 3:**
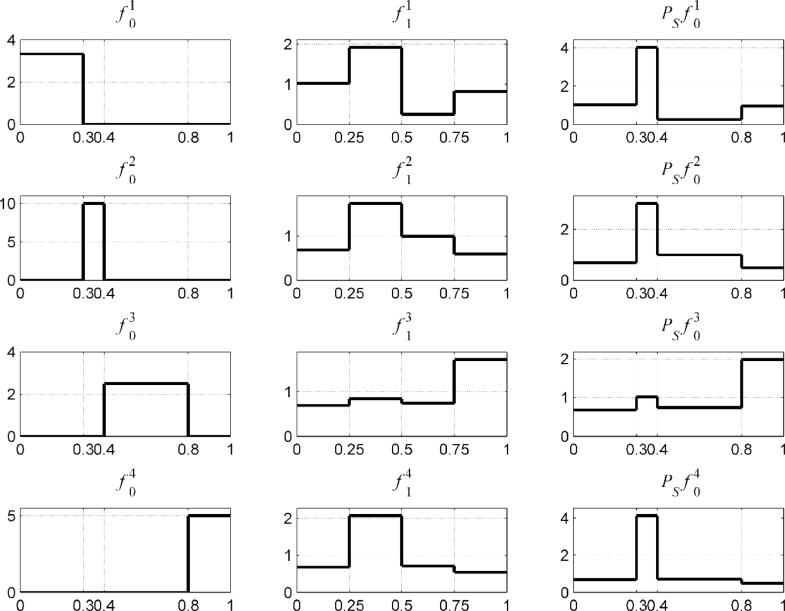
Example A: The density functions $f_{0}^{i}(x)$, $f_{1}^{i}(x)$ and $P_{s}f_{0}^{i}(x)$.

A set of initial densities $f_{0}^{i}$, i=1,…,4 over the partition ℜ is shown in Fig. 3. These are used to generate the set of initial states $X_{0}^{i}={\left\{x_{0,j}^{i}\right\}}_{j=1}^{5\times 10^{3}}$i=1,…,4. The corresponding final states $X_{1}^{i}={\left\{x_{1,j}^{i}\right\}}_{j=1}^{5\times 10^{3}}$, obtained by applying the map (64), were used to estimate piecewise constant densities $f_{1}^{i}$, i=1,…,4 over a uniform partition {[0, 0.25], (0.25, 0.5], (0.5, 0.75], (0.75, 1]} of [0, 1].

For this partition, the matrix ***D*** calculated using (29)

(66) $$D=\left[\begin{array}{cccc} 0.9921 & 0 & 0 & 0.0080\\ 0.2000 & 0.4000 & 0.4000 & 0\\ 0 & 0 & 1 & 0\\ 0.0079 & 0 & 0.2000 & 0.7920 \end{array}\right],$$

is non-singular.

The two stage approach detailed in Section 4, was used to estimate the matrix representation of the Frobenius–Perron operator associated with the deterministic transformation

(67) $$M=\left[\begin{array}{cccc} 0.3066 & 1.2094 & 0.0737 & 0.2880\\ 0.0685 & 0.3006 & 0.0989 & 0.0491\\ 0.2726 & 0.4091 & 0.2970 & 0.7926\\ 0.1363 & 0.8247 & 0.1419 & 0.0994 \end{array}\right].$$

Specifically, the constrained optimization problems (36), (37) and (39), (40) were solved using the lsqlin function in the Matlab Optimization Toolbox.

**Fig. 4:**
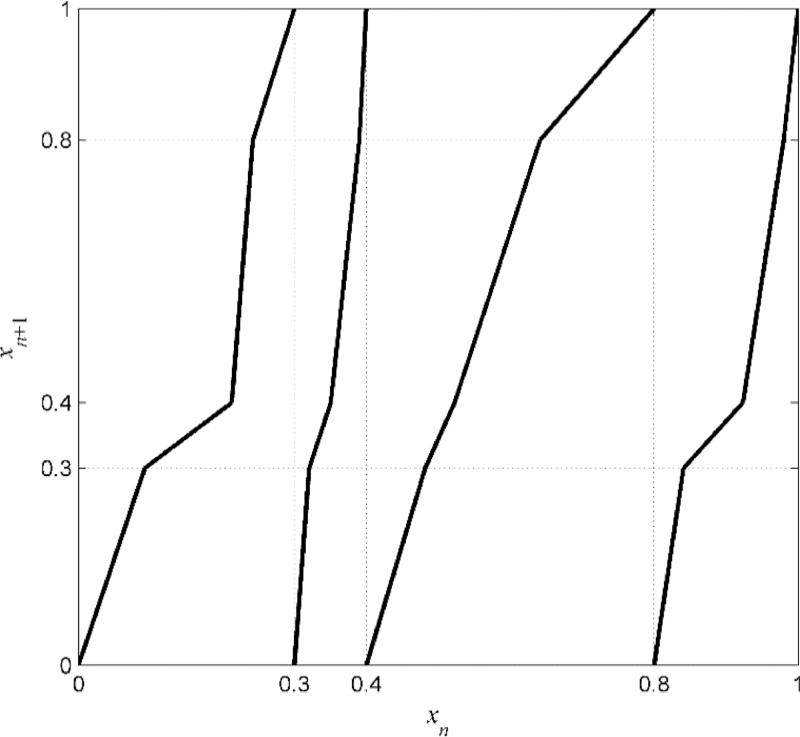
Example A: The identified transformation $\hat{S}$ of the underlying noisy system.

The reconstructed piecewise linear map $\hat{S}$ is shown in Fig. 4. The estimated coefficients of the identified piecewise linear semi-Markov transformation $\hat{S}{|}_{R_{i}}\left(x\right)={\hat{\alpha }}_{i,j}x+{\hat{\beta }}_{i,j}$ are ${\left({\hat{\alpha }}_{i,j}\right)}_{1\leq i,j\leq 4}=\left[\begin{array}{cccc} 3.26 & 0.83 & 13.57 & 3.47\\ 14.59 & 3.33 & 10.11 & 20.37\\ 3.67 & 2.44 & 3.37 & 1.26\\ 7.34 & 1.21 & 7.05 & 10.06 \end{array}\right],\;\;{\left({\hat{\beta }}_{i,j}\right)}_{1\leq i,j\leq 4}=\left[\begin{array}{cccc} 0 & 0.22 & -2.49 & -0.04\\ -4.38 & -0.77 & -3.15 & -7.15\\ -1.47 & -0.88 & -1.36 & -0.01\\ -5.87 & -0.72 & -6.11 & -9.06 \end{array}\right].$

**Fig. 5:**
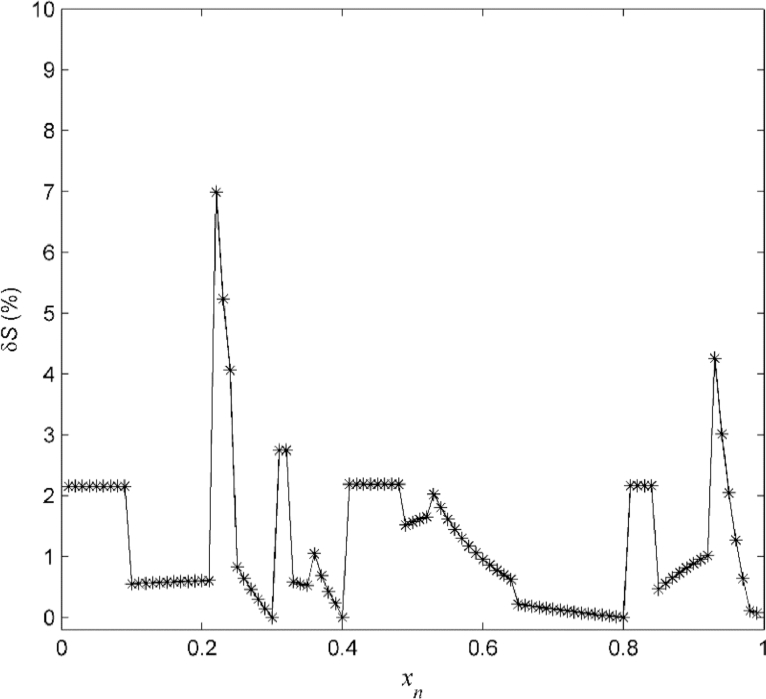
Example A: Relative error between the original map *S* and the identified map $\hat{S}$ evaluated for 99 uniformly spaced points.

The performance of the reconstruction algorithm is evaluated by computing the relative error

(68) $$\delta S\left(x\right)=100\left|\frac{S\left(x\right)-\hat{S}\left(x\right)}{S(x)}\right|,$$

for $x\in X_{ℜ}=\left\{0.01,\;0.02,\;\ldots ,\;0.99\right\}$, which is shown in Fig. 5. The performance of the new reconstruction algorithm for different levels of noise was compared with that of a previous algorithm [38] that does not incorporate knowledge of the noise density. Table 1 shows for comparison the *mean absolute percentage error* (MAPE)

(69) $$\text{MAPE}=\frac{100}{\theta_{\delta S}}\sum_{i=1}^{\theta_{\delta S}}\left|\frac{S\left(x_{i}\right)-\hat{S}\left(x_{i}\right)}{S(x_{i})}\right|,$$

for the two reconstruction approaches, where ${\left\{x_{i}\right\}}_{i=1}^{\theta_{\delta S}}=X_{ℜ}$. The results, clearly demonstrate the advantages of the new algorithm and in particular its robustness even for in the presence of very significant noise levels.

**Table 1:** Example A: Performance comparison between the new algorithm (A1) and the algorithm (A2) in [38] for different levels of noise.

$\eta =\sigma_{\xi }^{2}/\sigma_{x}^{2}$	0.0335	0.1621	0.9058	2.3638	4.0535	16.2140	22.6460
ɛ	0.02	0.04	0.10	0.15	0.20	0.40	0.50
MAPE^(A1)^	1.2171	1.4106	1.1222	0.3581	2.1578	2.2541	3.2035
MAPE^(A2)^	2.5362	3.1203	10.6281	34.2314	42.5629	56.2310	51.6851

### 6.2 Example B

**Fig. 6:**
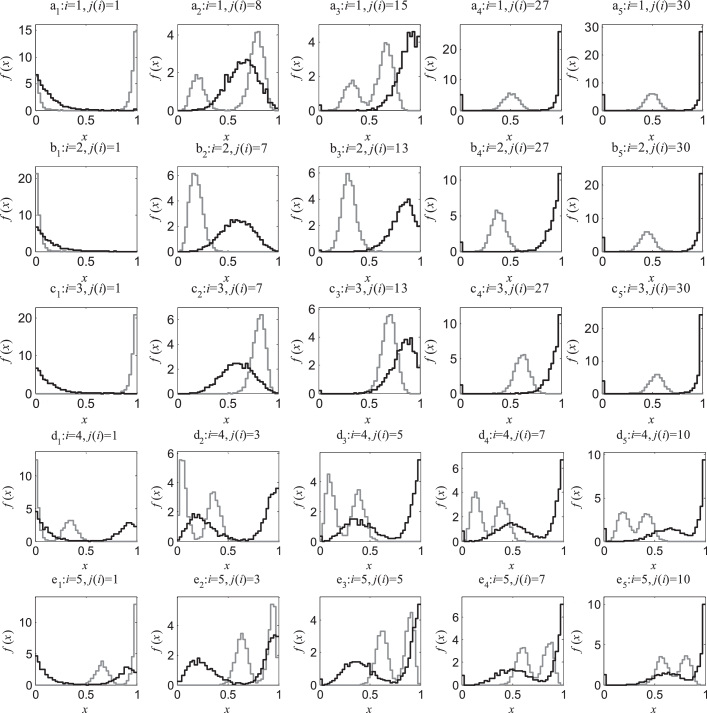
Example B: Examples of initial densities (gray lines) $f_{0,i}^{j(i)}$ and the corresponding final densities after one iteration (black lines) $f_{1,i}^{j(i)}$.

This example demonstrates the proposed algorithm to reconstruct a nonlinear continuous map. Specifically, we consider the logistic map defined by

(70) $$x_{n+1}=4x_{n}\left(1-x_{n}\right)+\xi_{n}\;\;\left(mod\;\;1\right)$$

where *ξ* is white noise following a Gaussian distribution function $N(0,\;{\left(5\times 10^{-3}\right)}^{2})$ truncated to the interval [−ɛ,ɛ] with ɛ=0.02. The aim is to infer a piecewise linear semi-Markov defined over a uniform partition ℜ with N=40 intervals, which approximates the logistic map *S*. The initial states $X_{0}^{i}={\left\{x_{0,j}^{i}\right\}}_{j=1}^{\theta }$, *i* = 1,…,100, $\theta =5\times 10^{3}$were generated by sampling a set of initial densities $f_{0,i}^{j(i)}$shown in Fig. 6 (see Appendix for more details). The corresponding final densities $f_{1,i}^{j(i)}$ over the uniform partition ℜ were estimated from $X_{1}^{i}={\left\{x_{i,j}\right\}}_{j=1}^{\theta }$, *i* = 1,…,100, the images under the noise perturbed transformation of the initial states $X_{0}^{i}={\left\{x_{0,j}^{i}\right\}}_{j=1}^{\theta }$ .

**Fig. 7:**
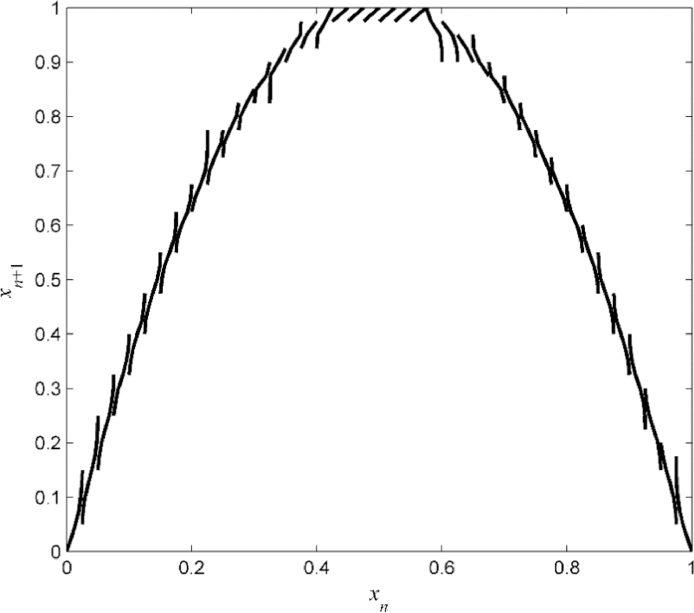
Example B: Reconstructed piecewise linear semi-Markov map *Ŝ* over the uniform partition $ℜ={\left\{R_{i}\right\}}_{i=1}^{40}$.

The approximate piecewise linear semi-Markov map, identified for *ε* = 0.02 using the algorithm in Section 5, is shown in Fig. 7.

**Fig. 8:**
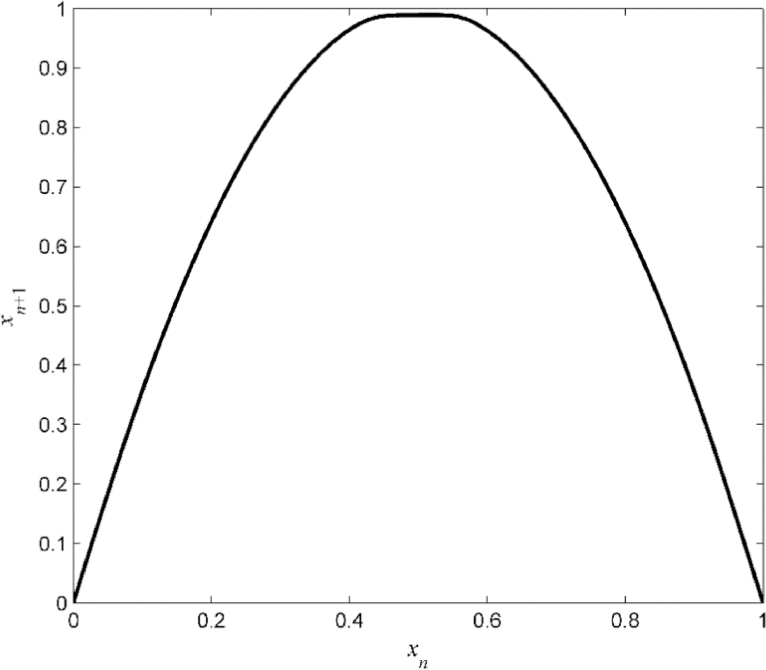
Example B: Identified smooth map $\bar{S}$ resulted from piecewise linear semi-Markov map in Fig. 7 with smoothing parameter 0.999.

**Fig. 9:**
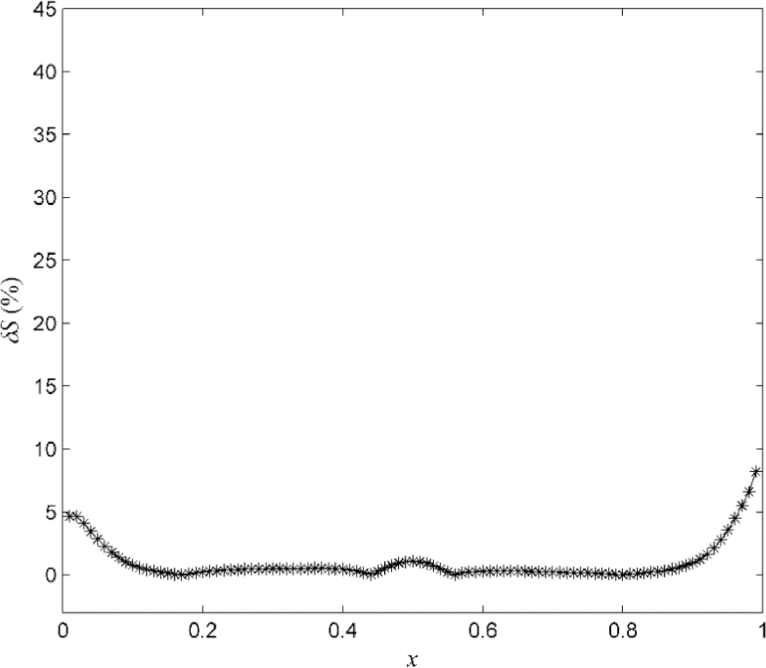
Example B: Relative error between the original map *S* and the identified smooth map $\bar{S}$ in Fig. 8 evaluated for 99 uniformly spaced points.

The smoothed map obtained with the smoothing parameter 0.999 is shown in Fig. 8, and the relative error calculated on the uniformly spaces points is shown in Fig. 9.

The *root mean square error* (RMSE) between the predicted density functions using original and identified maps calculated by

(71) $$\text{RMSE}=\sqrt{\frac{1}{N}\sum_{i=1}^{N}{\left(v_{i}-{\hat{v}}_{i}\right)}^{2}},$$

where ${\hat{v}}_{i}$ is the coefficient of predicted density function, is given in Table 2. As can be seen in Fig. 9 and Table 2, the approximation error has been very low. With the increase of the interval number, the reconstructed map is more close to the original one, and the stabilized distribution converges to the invariant density of the system, as proven in [22].

**Table 2:** Example B: RMSE between the predicted density functions *f_n_* using the identified map and those generated by the original noisy system.

*n*	1	2	3	5	10	20	50	100	200
RMSE	0.2887	0.2515	0.1915	0.2477	0.2133	0.2311	0.1608	0.2106	0.2120

**Fig. 10:**
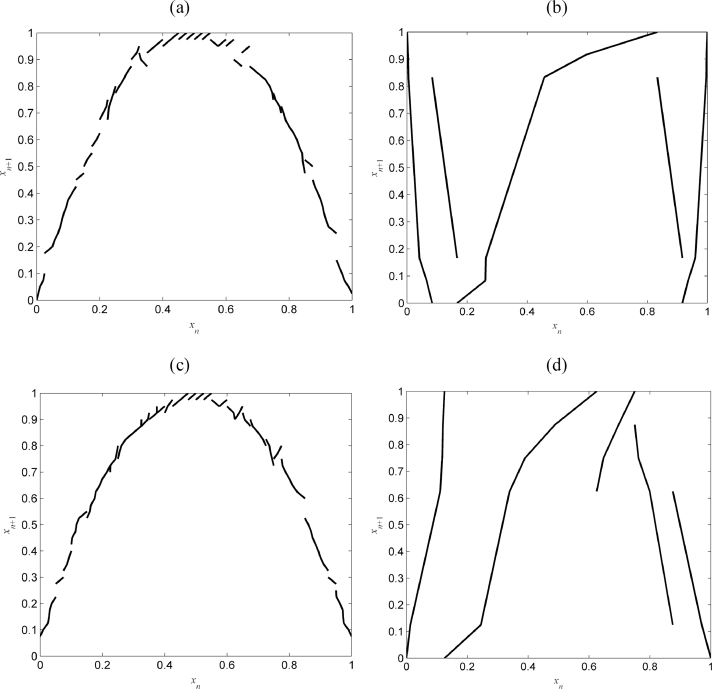
Example B: Reconstructed maps for noise level *ɛ* = 0.15 using (a) the new algorithm and (b) the algorithm in [38] and the reconstructed maps for noise *ɛ* = 0.50 using (c) the new algorithm and (d) the algorithm in [38].

As in the previous example, the performance of the new reconstruction method was compared with that of a previous method [38] for different levels of noise. The results are summarized in Table 3. As it can be seen, the new algorithm performs significantly and consistently better. This is also illustrated in Fig. 10 in which the maps reconstructed using the two algorithms for *ɛ* = 0.15 and *ɛ* = 0.50 are shown side-by-side. It is worth noting however, that one of the advantages of the original algorithm in [38] is that it includes an additional step to optimize the partition.

**Table 3:** Example B: Performance comparison between the new algorithm (A1) and the algorithm (A2) in [38] for different levels of noise.

$\eta =\sigma_{\xi }^{2}/\sigma_{x}^{2}$	0.0260	0.0978	0.5431	1.3692	3.5617	12.6023	17.6201
ɛ	0.02	0.04	0.10	0.15	0.20	0.40	0.50
MAPE^(A1)^	0.9282	0.9809	4.5791	3.1054	2.7850	4.6319	9.7981
MAPE^(A2)^	2.6120	2.9862	7.9236	78.3210	76.2536	58.1245	64.2101

## 7 Conclusions

This paper introduced a new method for reconstructing/approximating an unknown one-dimensional chaotic map that is perturbed by additive noise, from sequences of density functions. The emphasis here is on recovering the true transformation that generated the data rather than one of the many possible transformations that share the same invariant density functions.

By incorporating knowledge of the noise distribution, the new estimation method achieves dramatically better accuracy (i.e. over tenfold error reduction in some cases) for high levels of noise compared with a previous method that does not account for the noise density.

As highlighted in [38], it would be of interest to develop similar reconstruction approaches for higher-dimensional systems. The main challenge is to construct the transformation given the matrix-representation of the Frobenius–Perron operator [22].

## Appendix: Initial states for Example B in Section 6

The 100 sets of initial states used in the example are obtained by sampling the following density functions.

$$\begin{array}{ccc} f_{0,1}^{\beta_{1}}\left(x,\beta_{1}\right) & = & \frac{7}{10}\cdot \frac{x^{29}{\left(1-x\right)}^{\beta_{1}-1}}{B(30,\beta_{1})}+\frac{3}{10}\cdot \frac{x^{\beta_{1}-1}{\left(1-x\right)}^{29}}{B(\beta_{1},30)},\;\beta_{1}=1,2,\ldots ,30;\\ f_{0,2}^{\beta_{2}}\left(x,\beta_{2}\right) & = & \frac{x^{\beta_{2}-1}{\left(1-x\right)}^{29}}{B(\beta_{2},30)},\;\beta_{2}=1,2,\ldots ,25;\;f_{0,3}^{\beta_{3}}\left(x,\beta_{3}\right)=\frac{x^{29}{\left(1-x\right)}^{\beta_{3}-1}}{B(30,\beta_{3})},\;\beta_{3}=1,2,\ldots ,25;\\ f_{0,4}^{\beta_{4}}\left(x,\beta_{4}\right) & = & \frac{1}{2}\cdot \frac{x^{39}{\left(1-x\right)}^{\beta_{4}+19}}{B(40,\beta_{4})}+\frac{1}{2}\cdot \frac{x^{39}{\left(1-x\right)}^{\beta_{4}+19}}{B(40,\beta_{4})},\;\beta_{4}=1,2,\ldots ,10;\\ f_{0,5}^{\beta_{5}}\left(x,\beta_{5}\right) & = & \frac{1}{2}\cdot \frac{x^{\beta_{5}+19}{\left(1-x\right)}^{39}}{B(\beta_{5},40)}+\frac{1}{2}\cdot \frac{x^{\beta_{5}+19}{\left(1-x\right)}^{39}}{B(\beta_{5},40)},\;\beta_{5}=1,2,\ldots ,10; \end{array}$$

where B( ·, ·) is beta function.
